# Harmonization and Visualization of Data from a Transnational Multi-Sensor Personal Exposure Campaign

**DOI:** 10.3390/ijerph182111614

**Published:** 2021-11-04

**Authors:** Rok Novak, Ioannis Petridis, David Kocman, Johanna Amalia Robinson, Tjaša Kanduč, Dimitris Chapizanis, Spyros Karakitsios, Benjamin Flückiger, Danielle Vienneau, Ondřej Mikeš, Céline Degrendele, Ondřej Sáňka, Saul García Dos Santos-Alves, Thomas Maggos, Demetra Pardali, Asimina Stamatelopoulou, Dikaia Saraga, Marco Giovanni Persico, Jaideep Visave, Alberto Gotti, Dimosthenis Sarigiannis

**Affiliations:** 1 Department of Environmental Sciences, Jožef Stefan Institute, 1000 Ljubljana, Slovenia; david.kocman@ijs.si (D.K.); johanna.robinson@ijs.si (J.A.R.); tjasa.kanduc@ijs.si (T.K.); 2 Jožef Stefan International Postgraduate School, 1000 Ljubljana, Slovenia; 3 Environmental Engineering Laboratory, Department of Chemical Engineering, Aristotle University of Thessaloniki, 54124 Thessaloniki, Greece; ioannis.petridis89@gmail.com (I.P.); dimitris.chapizanis@gmail.com (D.C.); spyros.karakitsios@gmail.com (S.K.); denis@eng.auth.gr (D.S.); 4 HERACLES Research Centre on the Exposome and Health, Center for Interdisciplinary Research and Innovation, 54124 Thessaloniki, Greece; 5 Department of Epidemiology and Public Health, Swiss Tropical and Public Health Institute, CH-4051 Basel, Switzerland; benjamin.flueckiger@swisstph.ch (B.F.); danielle.vienneau@swisstph.ch (D.V.); 6 University of Basel, CH-4001 Basel, Switzerland; 7 RECETOX, Faculty of Science, Masaryk University, 62500 Brno, Czech Republic; ondrej.mikes@recetox.muni.cz (O.M.); celine.DEGRENDELE@univ-amu.fr (C.D.); ondrej.sanka@recetox.muni.cz (O.S.); 8 LCE, CNRS, Aix-Marseille University, 13003 Marseille, France; 9 Department of Atmospheric Pollution, National Environmental Health Centre, Institute of Health Carlos III, 28220 Madrid, Spain; sgarcia@isciii.es; 10 Atmospheric Chemistry and Innovative Technologies Laboratory, INRASTES, NCSR “Demokritos”, Aghia Paraskevi, 15310 Athens, Greece; tmaggos@ipta.demokritos.gr (T.M.); demetra.pard@gmail.com (D.P.); mina.stam@ipta.demokritos.gr (A.S.); dsaraga@ipta.demokritos.gr (D.S.); 11 Department of Science, Technology and Society, University School of Advanced Study IUSS, 27100 Pavia, Italy; marco.persico@iusspavia.it (M.G.P.); jaideep.visave@eucentre.it (J.V.); 12 Eucentre Foundation, Via A. Ferrata, 1, 27100 Pavia, Italy; gottial@gmail.com

**Keywords:** data fusion, multi-sensor, data visualization, data treatment, participant reports, air quality, exposure assessment

## Abstract

Use of a multi-sensor approach can provide citizens with holistic insights into the air quality of their immediate surroundings and their personal exposure to urban stressors. Our work, as part of the ICARUS H2020 project, which included over 600 participants from seven European cities, discusses the data fusion and harmonization of a diverse set of multi-sensor data streams to provide a comprehensive and understandable report for participants. Harmonizing the data streams identified issues with the sensor devices and protocols, such as non-uniform timestamps, data gaps, difficult data retrieval from commercial devices, and coarse activity data logging. Our process of data fusion and harmonization allowed us to automate visualizations and reports, and consequently provide each participant with a detailed individualized report. Results showed that a key solution was to streamline the code and speed up the process, which necessitated certain compromises in visualizing the data. A thought-out process of data fusion and harmonization of a diverse set of multi-sensor data streams considerably improved the quality and quantity of distilled data that a research participant received. Though automation considerably accelerated the production of the reports, manual and structured double checks are strongly recommended.

## 1. Introduction

The health impacts of poor air quality have become a central point of discussion in policy development and in personal exposure studies [1,2,3]. A growing selection of low-cost sensors (LCSs) that measure environmental conditions allow individuals to collect data about their own living environment and estimate their exposure to different stressors [4,5,6]. Several issues remain regarding bulkiness, design, power consumption, data loss [7], unreliable and (unintentionally) misleading data, lack of quality control, validation and calibration [8], and user experience [9]. Providing meaningful information to individuals about their environment and related stressors is in line with the United Nations Sustainable Development Goals (SDGs) calling for participatory, integrated, and sustainable human settlement planning (Target 11.3 [10]), which can only be achieved if the public is well-informed. Several goals and targets in the SDGs are assessed based on the “Mean urban air pollution of particulate matter (PM) of different sizes” indicator [11]. Considering the often-low spatial resolution of PM measurements (i.e., at a city level), typically only sampling outdoor air pollution, the use of individual low-cost PM sensors could be useful in estimating human exposure to PM.

Airborne particulate matter concentration is only one facet of air quality, and when assessing the impact of air quality on human health, pollutants such as nitrogen dioxide (NO_2_) [12], ozone (O_3_) [13], and volatile organic compounds (VOCs) [14,15] should be considered. Elevated levels of indoor carbon dioxide (CO_2_) concentrations can also pose health risks [16].

Data fusion techniques combine data from multiple source (i.e., sensors) and related information from databases to obtain more consistent, accurate, and useful information than can be obtained by the use of a single sensor alone, including fusing features and data to support decisions [17]. Fusing data from different low-cost sensors has previously been employed to supplement existing datasets from environmental monitoring networks with high-resolution spatiotemporal measurements from LCSs [18,19], by using mobile LCSs for air quality mapping in combination with dispersion model calculations [20] or by using stationary data with transport model results [21]. This enables the efficient integration of data derived from multiple sources at different stages of analysis and visualization.

An increase in the availability of devices with very diverse input parameters and data collection protocols poses some unique data fusion and visualization challenges, including non-standard timestamps, data gaps, different classifications, a multitude of data logging processes, etc. While LCSs generally provide a larger quantity of data, there is a lack of data on comparability from one device to another. Good metadata and documentation on how data are recorded and presented can help researchers make informed decisions and better comprehend potential issues prior to using the sensor [22]. The reliability and accuracy of LCSs may necessitate validation/calibration prior to use. Such processes are not standardized and can vary from device to device. The results are usually presented using the correlation coefficient, root mean square error, and mean absolute error, which, while useful, must be accompanied with information regarding the conditions under which the validation/calibration was performed [23]. In turn, this makes the process of data fusion and visualization more straightforward.

To facilitate data fusion and visualization, where one of the goals is to provide meaningful information to participants, there should be a greater focus on assessing the characteristics of the sensor itself, providing more context and associated uncertainties (where available) [22]. A benefit of participatory approaches, where citizens use LCSs, is the ability to gain additional (qualitative) information from the user through interviews or smartphone surveys [24] about specific environmental conditions to inform data fusion. Another benefit is the ability to obtain information about how well the sensors function.

Preparing visualizations of data for lay end users requires a balance in providing the most relevant data in an understandable way. Selecting the proper type of visualization can have a meaningful impact on the perception of the end user and the information that they are able to extract [25,26], and promote better risk assessment and reduction in exposure due to personal decision making [27]. An improvement, which is already being employed in some visualization efforts, is the ability of users to interact with the final dataset and make their own adjustments [28].

Collecting data from multi-sensor and multi-parameter data flows from hundreds of individuals involved in an exposure campaign produced unique issues and challenges which this paper specifically addresses. A key objective was to produce an aggregated and harmonized dataset that allowed for an efficient way of visualizing data via data fusion. Additionally, it provides a starting point for numerous individual-level and community-level exposure assessments and further data analysis, which will be explored in future research. An algorithm was developed that would clean, fuse, and visualize the collected data and present them to the participants in a straightforward and understandable report. This “final report” for the participants was generated in their respective local language, and included as much data as possible without making the report too long and complicated. The report aimed to provide enough details for participants to discern relevant information related to their local air quality, living environment, and behavior, with a view to eventually promote more environmental conscious lifestyles.

Specifically, the objectives of this study were to provide insights and specifics on:

- Outputs resulting from multi-sensor and multi-parameter data flows;
- Aggregation and harmonization of data collected;
- Production of tailored visualizations by fusing data from multiple sources, and automated compilation of individualized final reports.

## 2. Materials and Methods

**Figure 1:**
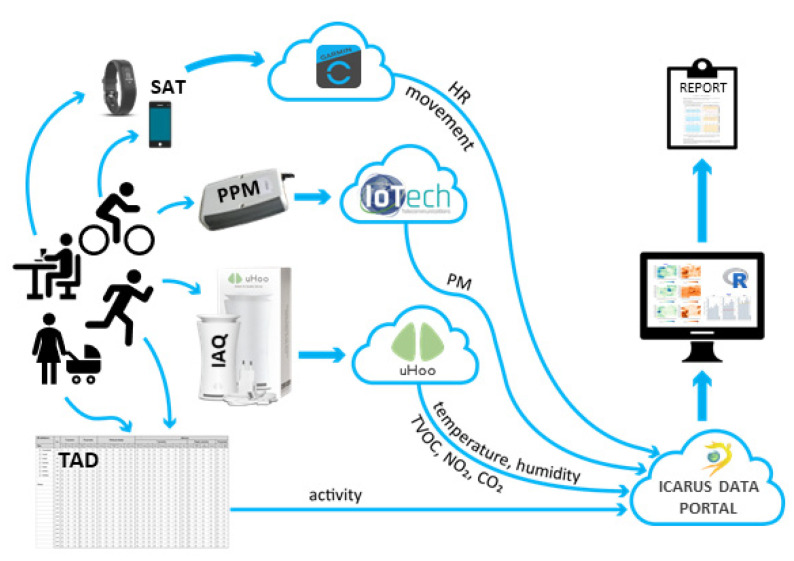
Schematic representation of data collection devices and protocols, transfer paths, aggregation, visualization, and delivery protocols.

Input data for data fusion and visualization were obtained from three sensor devices, data collected through questionnaires for households and individuals, and time activity diaries (TADs). They were part of the Integrated Climate forcing and Air pollution Reduction in Urban Systems (ICARUS) H2020 project, which applied integrated tools and strategies for urban impact assessment in support of air quality and climate change governance [29,30].For this purpose, about 100 participants were recruited in each of the seven selected European cities—Athens, Basel, Brno, Ljubljana, Madrid, Milano, and Thessaloniki—and were provided with all the tools required to collect the necessary data. The data were collected in two seasons in 2019—heating (winter) and non-heating (summer)—to observe any differences between the seasons, as the use of heating devices might influence air quality [31,32]. Two of the sensor devices were commercial: a smart activity tracker (SAT) and an indoor air quality (IAQ) sensing station. The third, called a personal particulate matter (PPM) sensing device, was specifically constructed for the purposes of the research project using the Arduino platform. A schematic representation of the devices and protocols used is shown in Figure 1. A detailed description of the campaign and its goals can be found in Robinson et al. [33]. All data cleaning, harmonization, fusion, visualization, and report compilation and output were done in R [34] with support from different R packages, e.g., ggplot2 [35], dplyr [36], knitr [37], and rmarkdown [38].

### 2.1. PPM Data

The PPM sensing device(IoTech Telecommunications, Thessaloniki, Greece) was designed for the purposes of the sampling campaign of the ICARUS project [39]. It collected PM concentration data in three class sizes (<1 µm (PM_1_), <2.5 µm (PM_2.5_), and <10 µm (PM_10_)) and ambient temperature as well as relative humidity data, in addition to GPS/location coordinates (including speed and altitude). As the device did not have a real-time clock (RTC) module (e.g., [40]), the timestamp was obtained by connecting it to an online server via a SIM card. Without this connection, the device did not provide data with accurate timestamps, which in turn produced several data gaps. Timestamp logging was irregular and inconsistent, as evident in an example of the dataset in section A of the Supplementary Data (SD-A).

### 2.2. SAT Data

A commercial SAT was used (Vivosmart 3, manufactured by Garmin, Olathe, Kansas, U.S. [41]) to collect heart rate and movement data with a minute resolution (e.g., average heart rate, stress level, sleep status, calories burned, etc.) As the export of data is not freely available through the Garmin interface, an additional connection between a dedicated ICARUS data portal and the Garmin Connect portal was established to transfer the data. The SAT data had very few gaps (excluding the time while the device was charging). Issues with data capture of heart rate occurred when the user did not fasten the wrist strap tight enough.

A brief overview of the SAT data was included in the final report as a summary table.

### 2.3. IAQ Data

A “uHoo Smart Indoor Air Quality (IAQ) sensor” (uHoo Limited, Singapore) [42], a stationary device with multiple sensors, was used in every household. At every full minute, the IAQ provided data on temperature, relative humidity, CO_2_, total VOCs (TVOCs), PM_2.5_, NO_2_, carbon monoxide (CO), ozone (O_3_), and air pressure.

Visualization of AQ parameters measured by the IAQ was limited to three parameters (CO_2_, NO_2_, and TVOCs) that showed the best performance during the collocation experiments with validated devices, as well as other tests. As offsets were observed for some sensors during these experiments, this specific dataset was visualized using a heatmap, focusing on relative changes in each variable over time. A heatmap, in this case, consists of tiles which are colored relative to all other tiles (lower values are lighter, higher values are darker), as implemented in Mahajan et al. [43]. Using minute values would create a heat map with small tiles, which would obscure the relative differences within a day. To counteract this, hourly values were calculated and used in the heat map, reducing the number of tiles from approximately 10,000 to 170.

### 2.4. ICARUS Data Portal

A dedicated data portal was constructed for the purposes of the ICARUS2020 project, and a decision support system (DSS) with it, which collected, compiled, and stored the data. The DSS additionally had a presentation tier with a user interface and a logic tier that stored the computational models and handled their execution [44]. In this study, the data portal was mainly utilized to store and obtain the PPM and SAT data in a uniform format, which allowed further manipulation and fusion of data.

### 2.5. TAD Data

A key data input was the TADs, which allowed the participant to record their activity, location, means of transport, and other variables for each hour of the day. These data were collected from each participant, for seven days in two seasons, for all cities, accumulating up to approximately 10,000 TADs.

There were two methods of filling in the TADs: one was to select only one option for each hour (i.e., majority activity) and the other was to allow participants to select multiple options within an hour. This posed a unique challenge in selecting which data point to use, which activity was more relevant or more characteristic for each hour.

Some manual corrections of the data were necessary in the final stages after observing some obvious mistakes in the recording of activity. As these corrections were not double-checked with the participants, only the most obvious mistakes were corrected, e.g., if a non-smoking person truly smoked in just one instance the entire period.

Because the data for activities were for hourly values and the sensor data had a minute resolution, the former was repeated 60 times per hour, which proved to be a major issue when calculating averages and trying to discern if there were meaningful differences between activities [45].

The TAD dataset was used in three visualizations, in combination with PPM and SAT data:

(a) A scatter plot was made for every PM size class and heart rate for both seasons. Additionally, the points were colored based on the activity at that minute, which allowed the reader to observe what activities took place at, for example, elevated levels of PM or elevated heart rate. Only the activities which the participant filled in were shown in the legend.
(b) A similar scatter plot as in (a) was constructed, with an additional layer which showed vertical bands or ribbons of different colors corresponding with the participant’s location and mode of transport. As this added another layer of complexity to the visualization, the decision was made to provide these plots only to specific individuals who expressed interest. Though activity information was missing in several TADs, the location and transport data were logged for almost the entire period of observation (for most participants). Consequently, participants could associate specific means of transport with elevated levels of PM, and corresponding activities with a higher heart rate.
(c) The third plot showed the average weekly PM values for each activity. Six plots were constructed, three per season, one for each PM size class.

TAD data were not used in combination with IAQ data due to the higher uncertainty associated with absolute values of CO_2_, NO_2_, and TVOCs.

### 2.6. Final Report Compilation and Production

The generation of final reports for participants was performed in three phases:

(a) Generation of plots as described in points 2.1.–2.4., which was followed for all of the participants. These plots were saved locally in a jpeg format and labeled according to each participant ID.
(b) Plots were integrated in a rmarkdown script, with the customization of each report designated in an Excel file. Each participant had a custom greeting with their name and gender-appropriate pronoun. All plots and other graphics were inserted using the include_graphics function in the knitr package.
(c) Finally, the script was iterated over all participants in a separate script to allow some further customizations. Some participants had additional visualizations (see 2.5 point b), while others had some omitted due to missing data. After all the reports were generated in the participants’ local language, they were manually checked for errors by local organizers in each participating city and distributed to all the participants.

In addition to the technical construction and production of the final report, the participant feedback and wishes for visualization were considered, where appropriate, by employing a user-centered approach and a structured focus group of participants [46].

### 2.7. Temporal Resolution and Data Treatment

A minute resolution of data was deemed as sufficient to provide enough detail of PM concentrations and exposure. The SAT and IAQ also logged data with a minute resolution, though these logs were at every full minute while the PPM sensing device logged the measurements at different fractions of the minute. These were later rounded to the nearest minute.

To compare the PM data with WHO guideline limits the minute resolution data were aggregated into daily means. More uncertainty was associated with PM daily means from the first and last day the participant was involved in the campaign, as the participants did not collect data for the entire 24 h period.

An outlier correction was performed for the PM data, where all values above 180 µg/m^3^ were set to 180 µg/m^3^, based on the maximum values provided by “Air quality in Europe” as part of the CITEAIR and CITEAIR II projects [47]. This approach was used only for visualizing the data and providing the final reports to participants for clearer data representation.

The PPM data showed good agreement with absolute values measured from a reference research-grade device, a GRIMM Model 11-A, and increasingly did so with larger time-averaging intervals [39].

## 3. Results and Discussion

### 3.1. A Merged Dataset

The final merged dataset had 93 columns. Due to sensor failures, data gaps, incorrect TAD filling, etc., there were several instances of empty columns or, in some cases, completely empty datasets per participant. This was appropriately labeled in the final reports.

Section B of the Supplementary Data (SD-B) presents an example of a completed dataset, with all the data harmonized to a 1 min resolution. Each dataset includes:

- Specific characteristics for each participant (age and gender);
- PPM data (PM values, temperature, humidity, battery charge level, location coordinates, speed, and altitude);
- SAT data (where several columns proved to be somewhat redundant and were therefore removed);
- IAQ data (which proved to be easiest to handle as they had a correct timestamp for each recorded value, almost no missing values, and a simple interface to download the data);
- TAD data, presented the same way as they were recorded on the physical paper sheets: location of the participant (home, office, indoor, outdoor), transport data (bus, car, foot, etc.), indoor and outdoor activities (cooking, smoking, sports, etc.), and some specific conditions for the indoor space the participant was in (burning candle or fireplace, open windows, and/or AC turned on).

### 3.2. Visualizing the Data

All the visualizations are presented and described here as they were shown in the final report to participants, and are collected as examples from different participants.

**Figure 2:**
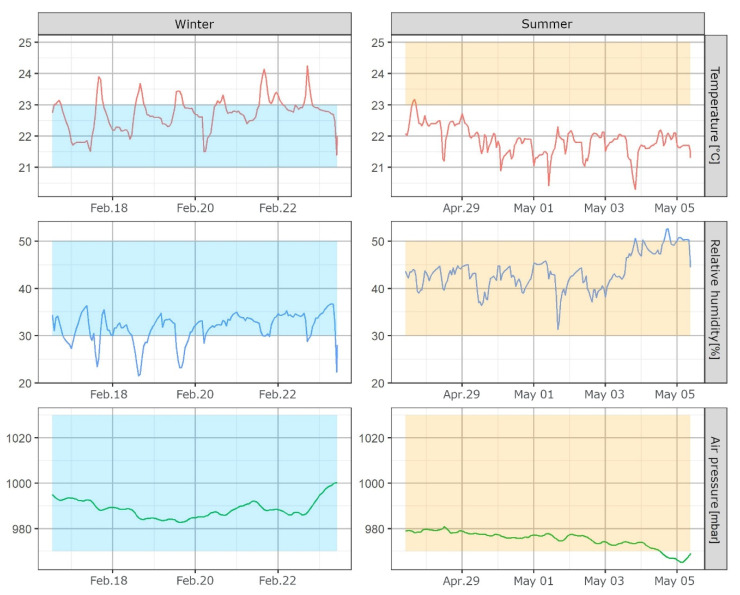
Faceted plots with meteorological variables—temperature, relative humidity, and air pressure; data from IAQ. Colored horizontal ribbons represent “optimal” values for each variable.

Figure 2 shows the temperature, relative humidity, and air pressure during both seasons (IAQ data). Non-heating and heating seasons are indicated as “Winter” and “Summer”, respectively, as the sampling campaign for all the cities extended from January to March 2019 for the heating season and from April to July 2019 for the non-heating season. The example visualizations are only from reports to participants from the city of Ljubljana, where the non-heating sampling campaign took place earlier than that in other cities and was therefore defined as summer. Meteorological data showed the highest accuracy when compared to reference instruments, and were in turn presented with absolute values. Although the ribbons show “optimal conditions” as per the general health and comfort guidelines (modified for the appropriate climate) [36], this information is somewhat subjective and can differ from person to person. As shown in the example in Figure 2, this person had very similar indoor temperatures in both seasons, and even though the summer values are mostly outside the “optimal zone”, one could argue that a constant temperature throughout the year provides more comfort to certain individuals.

Arranging the individual plots into columns according to season makes comparisons between the seasons easier.

**Figure 3:**
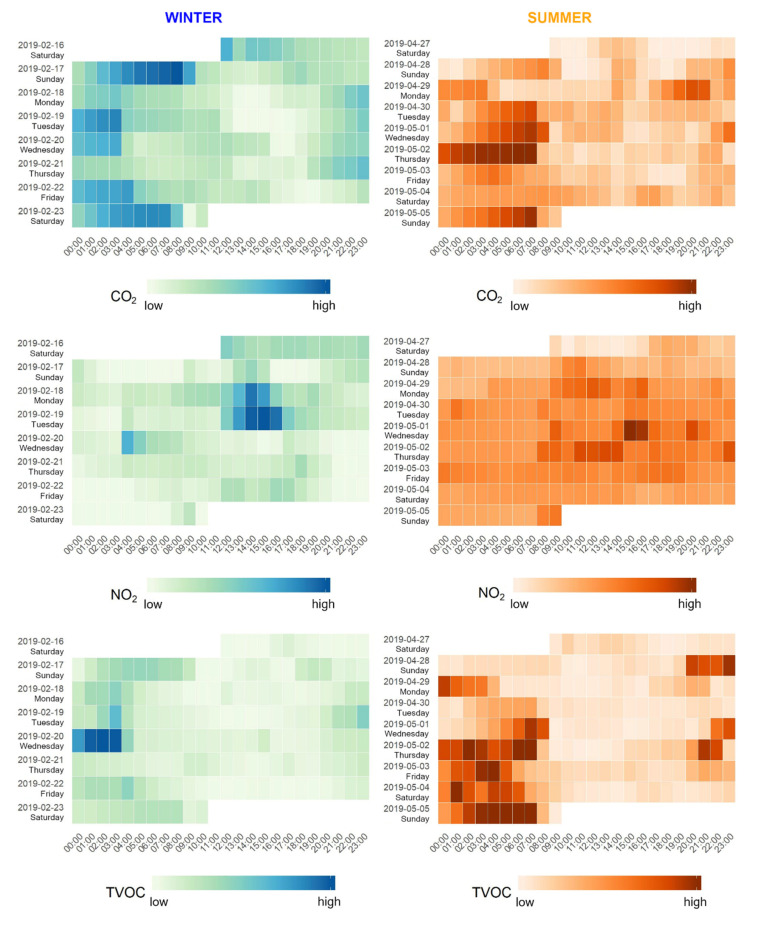
Faceted heatmaps of three pollutants (CO_2_, NO_2_, and TVOCs); data from IAQ.

Figure 3 shows an example of the compiled visualizations of CO_2_, NO_2_, and TVOCs for this particular household. We found that these parameters typically followed expected trends, e.g., decreased values of CO_2_ when opening a window and in turn increasing the NO_2_ values if it was in a high-traffic area [48], as seen in Figure 3 on Tuesday the 19th of February 2019 at around 13:00, when CO_2_ concentrations quickly fell and NO_2_ increased rapidly at the same time. The plots allow for an intuitive way of observing relative changes in these parameters by household. We used relative values in our reports, as collocation with a reference device has previously shown that the absolute values were not accurate enough to present to participants at that time [49], though newer research shows moderate to high correlation with reference instruments in laboratory conditions [50]. These relative values still give participants an insight into their indoor air quality and possible correlations with external factors such as traffic.

The layout of the visualization allows the reader to compare trends between seasons and between pollutants. For example, higher TVOC values during the evening and night could indicate poor ventilation in combination with a specific activity that raises the concentrations, such as cooking or smoking [51]. By putting these plots in the same figure, they can immediately observe the trends in the other two parameters and come to some conclusions.

Each date is also labeled with the written day of the week (language-specific) to facilitate better observation of specific trends.

**Figure 4:**
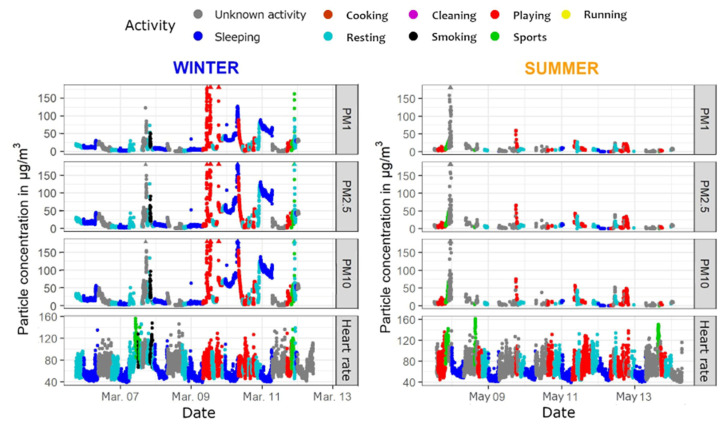
Three size classes of PM (PM_1_, PM_2.5_, and PM_10_) and heart rate values for both seasons with each point colored according to the associated activity; data from PPM, SAT, and TAD.

Figure 4 presents the concentrations of PM in three class sizes, heart rate, and designated activities for each minute during which the participant was involved in the data collection. Only the specific activities are shown; there is no additional information about the location of the participant, their mode of transport, or specific conditions in the household. Not including this information makes the visualizations less crowded and easier to read and understand, as determined by exchanges provided in a structured focus group [46]. All the values are also plotted with exact concentrations, given that the PPM device showed fairly accurate results compared to reference devices.

The participants could deduce by themselves some interpretations and extra information from the plots, e.g., a higher heart rate when running, dips during the night, a specific time of day when the PM concentrations were elevated and if they were perhaps related to a specific activity such as smoking or cooking, etc. This level of interpretation is only feasibly possible by the participants, because they would have a more complete overview of their surrounding and activities. To avoid the issue of recall, participants could be provided with their respective TADs.

No particular difficulties were encountered with constructing this visualization, with the possible exception of some alterations to the color scale and legend to also include the activities that the participant did not perform.

**Figure 5:**
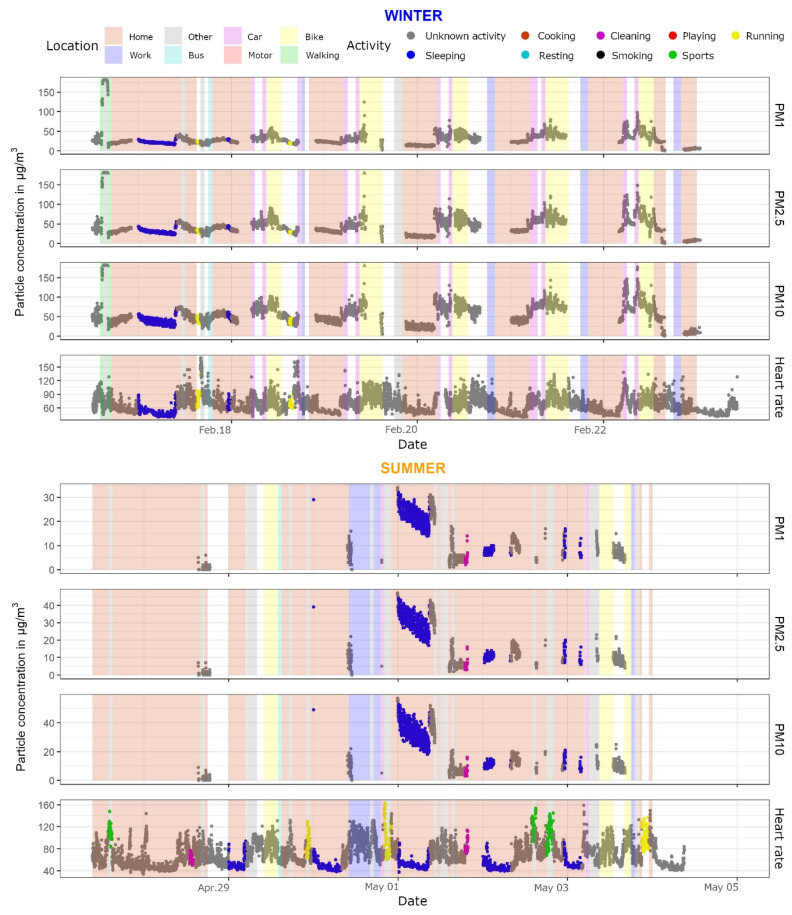
Three size classes of PM and heart rate values for both seasons with each point colored according to the associated activity and each ribbon representing a location or means of transport for that time period; data from PPM, SAT, and TAD.

An additional figure was created to include the location and transport of the participants, in addition to PM and activity values (Figure 5).

Several difficulties were encountered while constructing these plots, as the ribbons that show each activity needed a start and an end time for each location/transport at every interval. We considered only including a vertical line at each minute in the color of the location/transport, but this considerably increased the run-time per plot and was not efficient given the large number of plots to produce. An additional section of code was implemented to construct a separate data frame which had a start and an end time with a label for each location/transport. This was used in the ggplot2 geom_rect function while compiling the plot and noticeably reduced the time it took to compile each plot.

**Figure 6:**
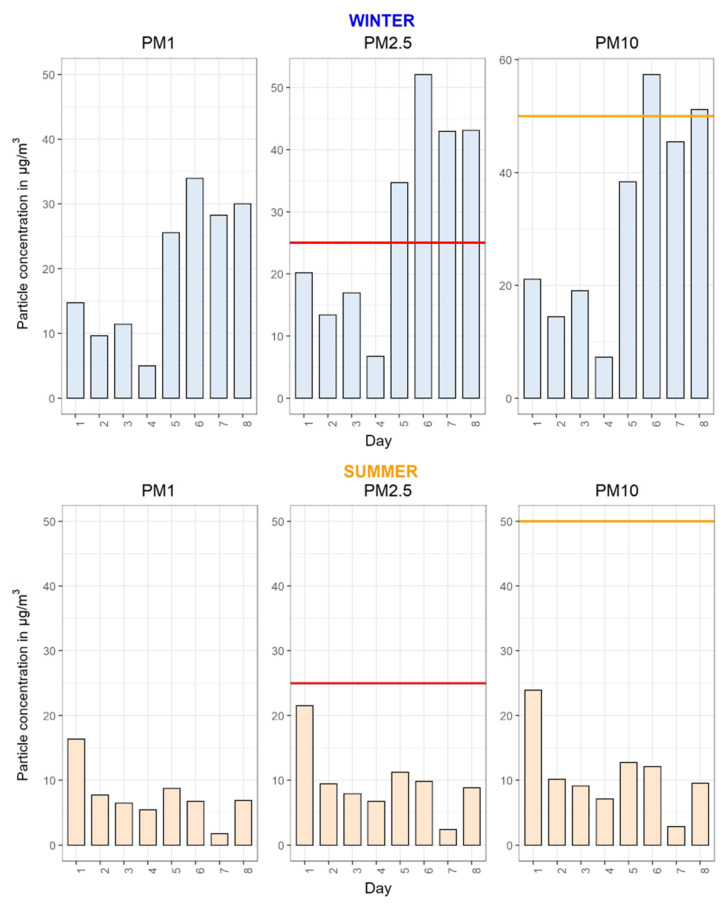
Faceted plots of average daily concentrations of three size classes of PM for each season, with WHO guidelines; data from PPM.

Figure 6 shows the daily average PM concentrations for both seasons, and is the only set of plots where guidelines or recommended values could be inserted. The WHO and the EU do not have minute or hourly guidelines for concentrations of PM, though studies show that short-term exposure to elevated levels can have adverse effects on health [52,53]. The WHO does provide daily guidelines for PM_2.5_ and PM_10_, which are 25 µg/m^3^ and 50 µg/m^3^, respectively [54], revised in 2021 to 15 µg/m^3^ and 45 µg/m^3^, respectively [55].

There are two important pieces of information in these plots, allowing the participant to observe (1) inter-seasonal differences and (2) day-to-day differences, while also having information about a specific size class of PM. This specific plot shows that the concentrations are generally higher in wintertime (more indoor activities, weather patterns that trap pollution in low-lying areas, combustion of solid fuels, more use of car/buses in contrast with cycling/walking, etc.), and when there are elevated levels of PM during the summer they are still much lower than in wintertime. The participant can also observe that some particular days have elevated levels of PM, which could be associated with specific activities performed that day (or weather patterns).

**Figure 7:**
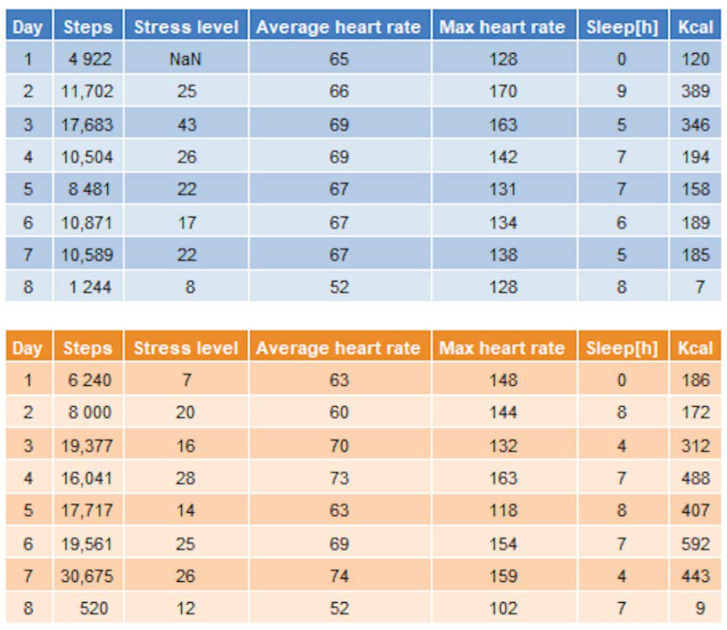
Aggregated data from SAT.

Figure 7 presents two tables showing the average values for each SAT variable for each day. No additional visualizations were made for the SAT data (apart from the heart rate plots in Figure 4 and Figure 5). There were several visualizations already available on the Garmin Connect portal for each variable.

**Figure 8:**
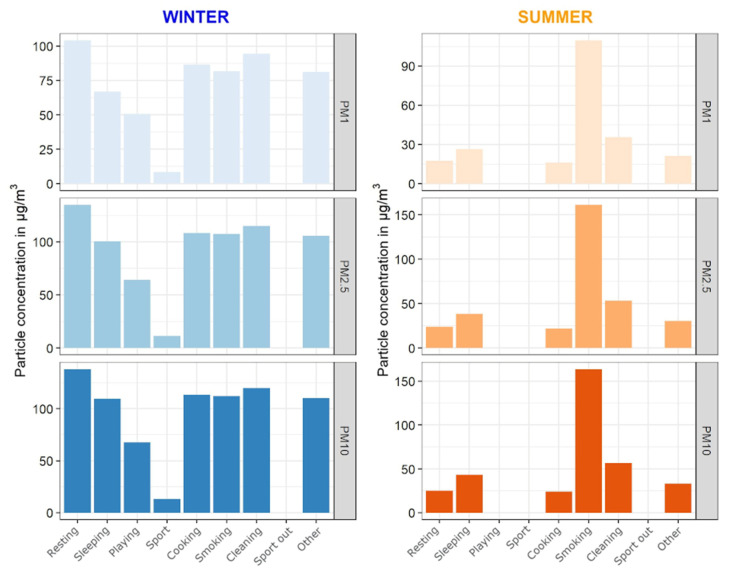
Faceted plots of average values of three size classes of PM (PM_1_, PM_2.5_, and PM_10_) for each specific activity and each season; data from PPM and TAD.

Figure 8 shows the average PM values for each activity as indicated by the participant in the TADs. There are certain shortcomings to this visualization as it does not provide any data about the number of instances for each activity, e.g., in this example there is only one hour of smoking indoors in the entire week during the summer season, but over 50 h of sleeping. Although the caption under the plots clearly states that the empty columns mean that there were no recorded instances of that specific activity, there can still be some confusion where the reader might assume that the average concentration is 0 µg/m^3^.

Primarily this plot should communicate differences between the activities in each respective reason. In the example provided in Figure 8, the PM values for smoking are higher than all other activities during the summer season, but not that different from all other activities during winter. A possible explanation would be that there is less natural air circulation during the winter (opening windows or doors), though there could be other explanations. This is another prime example where detailed information about their surroundings would give the individual the most accurate assessment of what the source of the elevated concentrations of PM could be.

### 3.3. The Final Report

An example of the final report provided to all participants is shown in section C of the Supplementary Data (SD-C). The report began with a personalized greeting, a general description of the project, and the contents of the report. There were also disclaimers about the nature of the low-cost sensors and the uncertainty associated with them. The next page (“Part A”) had a more detailed description of the study, the devices and approaches that were used, and what the reader should specifically focus on. “Part B” described the household conditions, focusing mainly on the data from the IAQ with Figure 2 and Figure 3, accompanied with appropriate captions.

“Part C” contained the plots concerning personal exposure to air pollutants, beginning with PM data, shown in Figure 4, Figure 5 and Figure 6. Figure 5 was provided only to a handful of participants who had more recorded data and requested a more thorough overview for the entire duration of their involvement. The physical activity information, shown in Figure 7, was presented next and accompanied with a more detailed description of each variable, including some measures for low, average, and elevated heart rate to aid the reader in their interpretation of the data. Figure 8, showing the average PM values for each activity, was included last, with a specific disclaimer that the scales on the *y*-axis are free.

Some general recommendations on “How to improve indoor air quality” were provided at the end of each report together with two tables extracted from the uHoo sensor device recommendations and descriptions [42].

### 3.4. Issues Faced and Recommendations for Future Studies

Several issues were encountered while compiling, cleaning, and visualizing the data collected from LCSs. While the PPM device proved to be the most accurate when compared with a reference instrument, it also had the most issues regarding data gaps and inconsistent timestamps. Two relatively small improvements to the device would have helped, as they would make the device independent of the GPRS signal: (a) installing a real-time clock (RTC) module which would provide consistent timestamps, and (b) increasing the internal storage and buffer to record PM values without a connection to the server. Several optimizations to reduce energy usage would be possible, e.g., less frequent GPS recordings while stationary, option to only upload the data when the device is charging, etc.

On the other hand, the IAQ had very consistent data streams, accurate timestamps, and a very intuitive interface. Two improvements would make the device function more independently: (a) a small internal storage for times when there was no wi-fi signal, which would allow the device to store the data in an internal buffer and upload it when the connection was re-established, and (b) a small battery to allow the device to function during power outages.

The SAT was very reliable, had an internal storage capacity for 14 days of data, and had a battery that lasted between 5 and 7 days. An improvement would be to provide a way to verify if the data is being logged correctly. At times, the device was not placed properly on the wrist or had some other error with data logging, and this was only observable at the end of the sampling campaign. Though the SAT did provide a uniform dataset it had to be extracted by a separate process in collaboration with the company that produced the device. Accessing data from commercial devices proved complicated and preconditioned on setting up exclusive deals with companies. Even when the deal is set, the entire data retrieval process is reliant on the cooperation of the company. It would be better if the raw data streams were open access.

A key improvement for the TADs would be to allow more granular activity logging during the day, e.g., every 15 min. TADs could also be somewhat customized to different participants or days of the week, e.g., participants who perform only one activity, such as work, during morning hours could have a different TAD during workdays than during weekends. The hourly resolution of TADs caused some issues when presenting and visualizing data for the participants as the average values were skewed, due to the fact that most activities do not have a duration of one hour nor do they start and end at full hours. This meant, for example, that someone who went for a 40 min run followed by smoking a cigarette might only have recorded “running” for that hour. Even though the person could have checked both activities, there would still be no information as to which part of that full hour the “smoking” vs. “running” occurred. Recording activities minute by minute would be a heavy burden for participants, so future research should focus on other solutions, such as complex activity recognition using machine learning, smartphones, or other tools [45,56].

Visualizing the data proved challenging at times and required unique solutions. The main challenge in producing the plots in Figure 2 proved to be the horizontal ribbon with “optimal values”, which had to be referenced in a way that would allow this value to be presented for each individual hour, while also enabling faceting of the plots. Additional variables with minimum and maximum data for each season were introduced, which shortened the script for the final construction of the plots.

A rather easy, though important, improvement for the plots in Figure 6 would be to show specific dates and days of the week instead of the number of days since the participant joined the sampling campaign. Participants do not always remember which day they started the campaign and would have to go back to the IAQ figure to find out. As a significant amount of time can elapse between the campaign and the distribution of reports to participants, it could be good for future studies to always indicate in the figures the date and day of the week.

Figure 8 could be improved by indicating the number of instances for each activity by coloring the bars according to a color scale reflecting the frequency of activities or by changing the width of each bar accordingly. The activities without data should be clearly marked with a symbol or a text. A requirement for a minimal amount of data should be considered to remove activities with only a few instances. The color schemes should also be intuitive, such as coloring winter blue (cold color), summer orange (warm color), or smoking black, which instinctively guides the reader.

Manually collected data from TADs were double-checked by the researchers as there were some non-obvious errors, e.g., smoking selected for a person who designated that they do not smoke, which sometimes indicated a user error and other times that the person was in fact an infrequent smoker. As with any dataset, these inconsistencies and all permutations can be very time-consuming to implement into the report-generating code. A large number of reports (and the associated data) also necessitates that there is a careful process when deciding what functions to use, and how much time and processing power they will need.

## 4. Conclusions

Data fusion and visualization of data obtained in personal exposure campaigns performed in seven European cities within the ICARUS project were conducted. By using a diverse set of devices (wearable and static, commercially available, and custom-made) with different temporal and spatial resolutions, a significant amount of data was obtained for each participant. Data fusion was performed in order to integrate the multi-sensor and multi-parameter datasets collected from the > 600 participants. Individualized reports were compiled for each participant with an automated process. Following these large-scale campaigns, several lessons were drawn and recommendations for future studies were provided.

Using low-cost sensors to assess air quality on an individual level presented some unique challenges, e.g., fusing data by rounding, duplicating, and removing certain parts of the timestamps, which allowed a uniform presentation on several plots. Mostly simple modifications were enough to provide some clarity and make data fusion more straightforward. Appropriate guidelines have to be considered carefully to avoid confusing the participant or giving false impressions on otherwise non-harmful concentrations of pollutants.

Participants should not be overwhelmed with the report, rather it should provide sufficient data for them to obtain as much meaningful information as possible. While the SAT provided a large amount of data, a decision was taken to only include the visualizations that were the most effective at communication. Apart from the number of visualizations, the appropriate type must also be carefully selected and curated. Our approach with relative values for NO_2_, CO_2,_ and TVOCs provided enough data to clearly see some trends, without providing unreliable absolute data values. On the other hand, the higher reliability and accuracy of PM concentrations and meteorological parameters enabled us to provide absolute values. A clear option should be included to observe trends between days, seasons, and activities. These visualizations must also reflect the results of collocations and validations made prior to deploying these devices. Citizens must be made aware of the accuracy (and shortcomings) of the device they are using and to what extent can they rely on the results. A properly structured report will guide them through the report itself and give them enough support to extract the most useful interpretation of the data that they can.

A well-informed public can collaborate with and react to changes in their environment, be it by influencing policy decisions or making changes to their individual lifestyles and behaviors. Using LCS provides a conduit for citizens to be empowered by data that they can collect, observe, and interpret. We, as researchers, must provide the necessary tools and options for them and guide them through the process. Changes in policy can come from the top-down or from the ground-up. In both cases, the citizens that are affected by these policy changes must be active participants in designing and implementing these solutions.

## Data Availability

Data available on request due to privacy restrictions.

## Supplementary Materials

The following are available online at https://www.mdpi.com/article/10.3390/ijerph182111614/s1 (SD-A: Example of timestamp gaps in PPM dataset; SD-B: Example of dataset used for visualizations and reports; and SD-C: Example of entire report for participants).
