# Supplementary material for: Harmonization and Visualization of Data from a Transnational Multi-Sensor Personal Exposure Campaign
Source: Int J Environ Res Public Health. 2021 Nov 4;18(21):11614. doi: 10.3390/ijerph182111614 (PMC8583633; doi:10.3390/ijerph182111614)
Supplement: Supplementary file 1 [file ijerph-18-11614-s001.zip › SD-A.pdf]

Supplementary data A (SD-A)

date	hour	pm10env	pm25env	pm100env	temp	humidity	vbatt	latitude	longitude	altitude	speed_kph	counter
19/05/09	11:50:34	4	4	5	249	326	79			270.299988	3.740000	4200
19/05/09	11:51:35	4	5	5	249	322	77			270.299988	3.740000	4260
19/05/09	11:52:34	3	4	4	249	346	81			270.299988	3.740000	4320
19/05/09	11:53:34	4	5	6	250	354	80			337.799988	1.130000	4380
19/05/09	11:54:34	4	5	6	250	354	76			371.399994	4.190000	4440
19/05/09	11:55:35	4	5	6	250	354	79			361.500000	9.110000	4500
19/05/09	11:56:35	3	4	4	250	354	81			349.000000	2.690000	4560
19/05/09	11:57:34	3	5	5	250	334	74			349.000000	2.690000	4620
19/05/09	11:58:33	2	3	4	249	373	79			349.000000	2.690000	4680
19/05/09	12:12:56	0	1	1	240	330	79			0.000000	0.000000	10
19/05/09	12:30:20	0	0	0	236	315	72			0.000000	0.000000	10
19/05/09	12:31:12	0	0	0	236	315	73			0.000000	0.000000	60
19/05/09	12:32:09	0	0	0	236	315	76			0.000000	0.000000	120
19/05/09	12:33:08	0	0	0	236	315	75			0.000000	0.000000	180
19/05/09	12:34:09	0	0	0	236	315	74			0.000000	0.000000	240
19/05/09	12:35:06	0	0	0	236	315	76			0.000000	0.000000	300
19/05/09	12:36:57	0	1	1	235	312	76			0.000000	0.000000	10
19/05/09	12:37:46	0	1	1	235	311	74			0.000000	0.000000	60
19/05/09	12:38:48	0	1	1	235	312	74			0.000000	0.000000	120
19/05/09	12:39:47	0	1	1	235	311	74			0.000000	0.000000	180
19/05/09	12:40:45	0	1	1	235	312	73			0.000000	0.000000	240
19/05/09	12:41:43	0	0	0	235	311	76			0.000000	0.000000	300
19/05/09	12:42:44	0	1	1	235	311	70			0.000000	0.000000	360
19/05/09	12:43:43	0	0	0	235	311	73			0.000000	0.000000	420
19/05/09	12:44:41	0	0	0	235	311	76			0.000000	0.000000	480
19/05/09	12:45:40	0	0	0	234	310	74			329.700012	4.280000	540
19/05/09	12:46:40	0	0	0	234	310	76			329.700012	4.280000	600
19/05/09	12:47:39	0	0	0	234	309	72			322.100006	1.520000	660
19/05/09	12:48:38	0	0	0	234	309	74			321.700012	0.720000	720
19/05/09	12:49:36	0	0	0	234	309	76			321.399994	0.720000	780
19/05/09	12:50:36	0	0	0	233	308	72			321.399994	0.720000	840
19/05/09	12:51:38	0	1	1	233	308	76			321.399994	0.720000	900
19/05/09	12:52:37	0	1	1	233	307	76			321.399994	0.720000	960
19/05/09	12:53:35	0	1	1	233	307	69			321.399994	0.720000	1020
19/05/09	12:54:34	0	0	0	233	307	73			321.399994	0.720000	1080
19/05/09	12:55:33	0	0	0	233	307	74			321.399994	0.720000	1140
19/05/09	12:56:33	0	0	0	233	306	76			329.899994	1.610000	1200
19/05/09	12:57:35	0	0	0	232	306	75			329.899994	1.610000	1260
19/05/09	12:58:36	0	0	0	233	306	73			295.700012	3.300000	1320
19/05/09	12:59:35	0	0	0	232	306	75			295.700012	3.300000	1380
19/05/09	13:00:33	5	7	7	231	337	74			295.700012	3.300000	1440
19/05/09	13:01:32	3	5	5	229	364	76			295.700012	3.300000	1500
19/05/09	13:02:31	3	4	4	229	396	73			295.700012	3.300000	1560
19/05/09	13:03:30	4	5	5	228	409	73			295.700012	3.300000	1620

DATA HIDDEN
