# Supplementary material for: Harmonization and Visualization of Data from a Transnational Multi-Sensor Personal Exposure Campaign
Source: Int J Environ Res Public Health. 2021 Nov 4;18(21):11614. doi: 10.3390/ijerph182111614 (PMC8583633; doi:10.3390/ijerph182111614)
Supplement: Supplementary file 1 [file ijerph-18-11614-s001.zip › SD-B.pdf]

				TAD														SAT					
PID	DATE	DAY NUMBER	TIME	LOCATION	DETAILS	ACTIVITY IN1	ACTIVITY IN2	ACTIVITY IN3	ACTIVITY IN4	ACTIVITY IN5	ACTIVITY IN6	ACTIVITY IN7	ACTIVITY OUT	HOUSE CONDITIONS WINDOWS	HOUSE CONDITIONS AC/FAN	HOUSE CONDITIONS CANDLE	HOUSE CONDITIONS FIREPLACE	AVG HEART RATE	STEPS	DISTANCE (M)	KCAL	M.E.T	STRESS
LJU_S_P007	02/05/2019	6	07:47	indoors	Home	NA	Sleep	NA	NA	NA	NA	NA	NA	NA	NA	NA	NA	44	0	0	0	0.067	2
LJU_S_P007	02/05/2019	6	07:48	indoors	Home	NA	Sleep	NA	NA	NA	NA	NA	NA	NA	NA	NA	NA	42	0	0	0	0.067	1
LJU_S_P007	02/05/2019	6	07:49	indoors	Home	NA	Sleep	NA	NA	NA	NA	NA	NA	NA	NA	NA	NA	41	0	0	0	0.067	1
LJU_S_P007	02/05/2019	6	07:50	indoors	Home	NA	Sleep	NA	NA	NA	NA	NA	NA	NA	NA	NA	NA	46	0	0	0	0.067	1
LJU_S_P007	02/05/2019	6	07:51	indoors	Home	NA	Sleep	NA	NA	NA	NA	NA	NA	NA	NA	NA	NA	43	0	0	0	0.067	2
LJU_S_P007	02/05/2019	6	07:52	indoors	Home	NA	Sleep	NA	NA	NA	NA	NA	NA	NA	NA	NA	NA	43	0	0	0	0.067	2
LJU_S_P007	02/05/2019	6	07:53	indoors	Home	NA	Sleep	NA	NA	NA	NA	NA	NA	NA	NA	NA	NA	43	0	0	0	0.067	2
LJU_S_P007	02/05/2019	6	07:54	indoors	Home	NA	Sleep	NA	NA	NA	NA	NA	NA	NA	NA	NA	NA	44	0	0	0	0.067	2
LJU_S_P007	02/05/2019	6	07:55	indoors	Home	NA	Sleep	NA	NA	NA	NA	NA	NA	NA	NA	NA	NA	45	0	0	0	0.067	2
LJU_S_P007	02/05/2019	6	07:56	indoors	Home	NA	Sleep	NA	NA	NA	NA	NA	NA	NA	NA	NA	NA	43	0	0	0	0.067	2
LJU_S_P007	02/05/2019	6	07:57	indoors	Home	NA	Sleep	NA	NA	NA	NA	NA	NA	NA	NA	NA	NA	44	0	0	0	0.067	1
LJU_S_P007	02/05/2019	6	07:58	indoors	Home	NA	Sleep	NA	NA	NA	NA	NA	NA	NA	NA	NA	NA	43	0	0	0	0.067	1
LJU_S_P007	02/05/2019	6	07:59	indoors	Home	NA	Sleep	NA	NA	NA	NA	NA	NA	NA	NA	NA	NA	43	0	0	0	0.067	1
LJU_S_P007	02/05/2019	6	08:00	indoors	Home	NA	NA	NA	NA	NA	NA	NA	NA	Open Window	NA	NA	NA	42	0	0	0	0.067	24
LJU_S_P007	02/05/2019	6	08:01	indoors	Home	NA	NA	NA	NA	NA	NA	NA	NA	Open Window	NA	NA	NA	54	0	0	0	0.067	24
LJU_S_P007	02/05/2019	6	08:02	indoors	Home	NA	NA	NA	NA	NA	NA	NA	NA	Open Window	NA	NA	NA	89	0	0	0	0.067	24
LJU_S_P007	02/05/2019	6	08:03	indoors	Home	NA	NA	NA	NA	NA	NA	NA	NA	Open Window	NA	NA	NA	52	0	0	0	0.067	3
LJU_S_P007	02/05/2019	6	08:04	indoors	Home	NA	NA	NA	NA	NA	NA	NA	NA	Open Window	NA	NA	NA	40	0	0	0	0.067	3
LJU_S_P007	02/05/2019	6	08:05	indoors	Home	NA	NA	NA	NA	NA	NA	NA	NA	Open Window	NA	NA	NA	40	0	0	0	0.067	3
LJU_S_P007	02/05/2019	6	08:06	indoors	Home	NA	NA	NA	NA	NA	NA	NA	NA	Open Window	NA	NA	NA	40	0	0	0	0.067	2
LJU_S_P007	02/05/2019	6	08:07	indoors	Home	NA	NA	NA	NA	NA	NA	NA	NA	Open Window	NA	NA	NA	40	0	0	0	0.067	2
LJU_S_P007	02/05/2019	6	08:08	indoors	Home	NA	NA	NA	NA	NA	NA	NA	NA	Open Window	NA	NA	NA	40	0	0	0	0.067	2
LJU_S_P007	02/05/2019	6	08:09	indoors	Home	NA	NA	NA	NA	NA	NA	NA	NA	Open Window	NA	NA	NA	42	0	0	0	0.067	2
LJU_S_P007	02/05/2019	6	08:10	indoors	Home	NA	NA	NA	NA	NA	NA	NA	NA	Open Window	NA	NA	NA	41	0	0	0	0.067	2
LJU_S_P007	02/05/2019	6	08:11	indoors	Home	NA	NA	NA	NA	NA	NA	NA	NA	Open Window	NA	NA	NA	40	0	0	0	0.067	2
LJU_S_P007	02/05/2019	6	08:12	indoors	Home	NA	NA	NA	NA	NA	NA	NA	NA	Open Window	NA	NA	NA	45	0	0	0	0.067	3
LJU_S_P007	02/05/2019	6	08:13	indoors	Home	NA	NA	NA	NA	NA	NA	NA	NA	Open Window	NA	NA	NA	44	0	0	0	0.067	3
LJU_S_P007	02/05/2019	6	08:14	indoors	Home	NA	NA	NA	NA	NA	NA	NA	NA	Open Window	NA	NA	NA	42	0	0	0	0.067	3
LJU_S_P007	02/05/2019	6	08:15	indoors	Home	NA	NA	NA	NA	NA	NA	NA	NA	Open Window	NA	NA	NA	51	0	0	0	0.067	2
LJU_S_P007	02/05/2019	6	08:16	indoors	Home	NA	NA	NA	NA	NA	NA	NA	NA	Open Window	NA	NA	NA	43	0	0	0	0.067	2
LJU_S_P007	02/05/2019	6	08:17	indoors	Home	NA	NA	NA	NA	NA	NA	NA	NA	Open Window	NA	NA	NA	44	0	0	0	0.067	2
LJU_S_P007	02/05/2019	6	08:18	indoors	Home	NA	NA	NA	NA	NA	NA	NA	NA	Open Window	NA	NA	NA	44	0	0	0	0.067	2
LJU_S_P007	02/05/2019	6	08:19	indoors	Home	NA	NA	NA	NA	NA	NA	NA	NA	Open Window	NA	NA	NA	44	0	0	0	0.067	2
LJU_S_P007	02/05/2019	6	08:20	indoors	Home	NA	NA	NA	NA	NA	NA	NA	NA	Open Window	NA	NA	NA	45	0	0	0	0.067	2
LJU_S_P007	02/05/2019	6	08:21	indoors	Home	NA	NA	NA	NA	NA	NA	NA	NA	Open Window	NA	NA	NA	43	0	0	0	0.067	3
LJU_S_P007	02/05/2019	6	08:22	indoors	Home	NA	NA	NA	NA	NA	NA	NA	NA	Open Window	NA	NA	NA	43	0	0	0	0.067	3
LJU_S_P007	02/05/2019	6	08:23	indoors	Home	NA	NA	NA	NA	NA	NA	NA	NA	Open Window	NA	NA	NA	43	0	0	0	0.067	3
LJU_S_P007	02/05/2019	6	08:24	indoors	Home	NA	NA	NA	NA	NA	NA	NA	NA	Open Window	NA	NA	NA	48	0	0	0	0.067	2
LJU_S_P007	02/05/2019	6	08:25	indoors	Home	NA	NA	NA	NA	NA	NA	NA	NA	Open Window	NA	NA	NA	44	0	0	0	0.067	2
LJU_S_P007	02/05/2019	6	08:26	indoors	Home	NA	NA	NA	NA	NA	NA	NA	NA	Open Window	NA	NA	NA	43	0	0	0	0.067	2
LJU_S_P007	02/05/2019	6	08:27	indoors	Home	NA	NA	NA	NA	NA	NA	NA	NA	Open Window	NA	NA	NA	44	0	0	0	0.067	2
LJU_S_P007	02/05/2019	6	08:28	indoors	Home	NA	NA	NA	NA	NA	NA	NA	NA	Open Window	NA	NA	NA	43	0	0	0	0.067	2
LJU_S_P007	02/05/2019	6	08:29	indoors	Home	NA	NA	NA	NA	NA	NA	NA	NA	Open Window	NA	NA	NA	44	0	0	0	0.067	2
LJU_S_P007	02/05/2019	6	08:30	indoors	Home	NA	NA	NA	NA	NA	NA	NA	NA	Open Window	NA	NA	NA	45	0	0	0	0.067	2
LJU_S_P007	02/05/2019	6	08:31	indoors	Home	NA	NA	NA	NA	NA	NA	NA	NA	Open Window	NA	NA	NA	43	0	0	0	0.067	2
LJU_S_P007	02/05/2019	6	08:32	indoors	Home	NA	NA	NA	NA	NA	NA	NA	NA	Open Window	NA	NA	NA	43	0	0	0	0.067	2
LJU_S_P007	02/05/2019	6	08:33	indoors	Home	NA	NA	NA	NA	NA	NA	NA	NA	Open Window	NA	NA	NA	44	0	0	0	0.067	4
LJU_S_P007	02/05/2019	6	08:34	indoors	Home	NA	NA	NA	NA	NA	NA	NA	NA	Open Window	NA	NA	NA	43	0	0	0	0.067	4

PPM											IAQ												
PM10ENV	PM25ENV	PM100ENV	TEMP	HUMIDITY	VBATT	LATITUDE	LONGITUDE	SPEED KPH	ALTITUDE	COUNTER	TEMPERATURE	RELATIVE HUMIDITY	PM2.5	TVOC	CO2	CO	AIR PRESSURE	OZONE	NO2	AGE	GENDER	city	Season
7	8	10	24.6	29	98	0	0	0	0	10	21.8	44.44	26.14	1156	1649	0	977.01	9.69	32.7	35	male	LJU	S
7	8	10	24.5	29	99	0	0	0	0	60	21.8	44.48	19.14	1156	1679	0	976.96	9.25	26.1	35	male	LJU	S
7	9	10	24.5	29	99	0	0	0	0	120	21.8	44.44	20.1	1156	1682	0	976.93	9.69	32.7	35	male	LJU	S
7	9	10	24.5	29	98	0	0	0	0	180	21.8	44.42	29.41	1156	1624	0	976.91	9.68	32.6	35	male	LJU	S
7	9	10	24.5	29	99	0	0	0	0	240	21.8	44.43	36.2	1156	1647	0	976.94	9.69	32.8	35	male	LJU	S
9	11	11	24.5	29	98	0	0	0	0	300	21.8	44.43	18.13	1156	1613	0	976.93	9.67	32.5	35	male	LJU	S
8	10	14	24.6	28.9	98	0	0	0	0	360	21.8	44.58	18.59	1156	1541	0	976.9	9.68	32.6	35	male	LJU	S
8	10	10	24.6	29	98	0	0	0	0	420	21.8	44.44	23.8	1156	1599	0	976.93	9.7	32.9	35	male	LJU	S
9	11	11	24.6	29	98	0	0	0	0	480	21.8	44.54	43.77	1156	1654	0	976.88	9.7	32.9	35	male	LJU	S
7	9	9	24.6	28.9	98	0	0	0	0	540	21.8	44.52	27.26	1156	1660	0	976.87	9.78	34.1	35	male	LJU	S
7	9	9	24.6	28.9	98	0	0	0	0	600	21.8	44.5	17.09	1156	1658	0	976.82	9.78	34.2	35	male	LJU	S
8	9	9	24.6	29	98	0	0	0	0	660	21.8	44.53	17.16	1156	1671	0	976.87	9.7	32.9	35	male	LJU	S
6	8	9	24.6	29	98	0	0	0	0	720	21.8	44.47	23.06	1156	1686	0	976.86	9.68	32.6	35	male	LJU	S
9	10	11	24.6	28.9	99	0	0	0	0	780	21.8	44.43	35.31	1156	1628	0	976.86	9.66	32.3	35	male	LJU	S
8	9	10	24.6	28.9	99	0	0	0	0	840	21.8	44.52	25.14	1156	1662	0	976.83	10.39	43.3	35	male	LJU	S
9	11	11	24.6	28.9	99	0	0	0	0	900	21.8	44.81	19.37	1156	1734	0	976.85	9.66	32.2	35	male	LJU	S
9	11	11	24.6	28.9	99	0	0	0	0	960	21.8	44.72	20.02	1156	1696	0	976.9	9.68	32.4	35	male	LJU	S
8	9	9	24.6	29	99	0	0	0	0	1020	21.8	43.3	35.18	1156	1658	0	976.92	9.84	35.8	35	male	LJU	S
8	11	12	24.6	28.9	99	0	0	0	0	1080	21.7	41.92	32.49	1156	1407	0	976.93	10.03	39.8	35	male	LJU	S
7	8	8	24.6	28.8	99	0	0	0	0	1140	21.7	41.42	20.27	1156	1055	0	976.91	10.17	42.3	35	male	LJU	S
11	13	15	24.6	28.4	99	0	0	0	0	1200	21.7	41.26	56.36	1098	851	0	976.84	10.25	43.6	35	male	LJU	S
13	15	16	24.6	27.9	99	0	0	0	0	1260	21.6	41.18	25.74	935	797	0	976.9	10.3	44.5	35	male	LJU	S
13	15	16	24.6	27.9	99	0	0	0	0	1320	21.5	41.13	42.17	791	821	0	976.87	10.33	45	35	male	LJU	S
14	18	19	24.5	27.4	98	0	0	0	0	1380	21.5	41.21	21.63	665	738	0	976.86	10.37	45.6	35	male	LJU	S
12	16	16	24.4	27.2	99	0	0	0	0	1440	21.5	41.08	20.69	567	708	0	976.9	10.41	46.4	35	male	LJU	S
14	19	20	24.4	27.2	99	0	0	0	0	1500	21.5	40.91	42.31	471	722	0	976.83	10.48	47.5	35	male	LJU	S
14	17	18	24.4	27.1	97	0	0	0	0	1560	21.4	40.86	41.04	383	680	0	976.84	10.51	48	35	male	LJU	S
13	17	19	24.3	27	98	0	0	0	0	1620	21.4	40.8	37.63	304	611	0	976.87	10.53	48.4	35	male	LJU	S
13	17	19	24.3	27	99	0	0	0	0	1680	21.3	40.84	23.43	239	606	0	976.91	10.57	49	35	male	LJU	S
13	17	18	24.3	26.8	98	0	0	0	0	1740	21.3	40.8	32.85	199	636	0	976.87	10.59	49.3	35	male	LJU	S
12	16	17	24.2	26.8	99	0	0	0	0	1800	21.3	40.65	23.34	177	543	0	976.9	10.6	49.6	35	male	LJU	S
14	16	16	24.2	26.8	98	0	0	0	0	1860	21.3	40.7	33.21	150	516	0	976.82	10.62	49.8	35	male	LJU	S
12	15	15	24.2	26.8	99	0	0	0	0	1920	21.2	40.78	29.63	139	575	0	976.85	10.64	50.2	35	male	LJU	S
14	18	18	24.1	26.7	99	0	0	0	0	1980	21.2	40.71	30.98	124	575	0	976.91	10.63	50	35	male	LJU	S
14	18	18	24.1	26.7	99	0	0	0	0	2040	21.2	40.6	19.53	108	538	0	976.89	10.68	50.9	35	male	LJU	S
14	17	18	24.1	26.7	99	0	0	0	0	2100	21.2	40.61	22.25	93	565	0	976.9	10.71	51.4	35	male	LJU	S
13	19	20	24	26.7	99	0	0	0	0	2160	21.1	40.6	46.32	82	598	0	976.92	10.73	51.6	35	male	LJU	S
14	18	18	24	26.7	99	0	0	0	0	2220	21.1	40.47	27.16	71	600	0	976.92	10.72	51.6	35	male	LJU	S
12	13	13	24	26.7	99	0	0	0	0	2280	21.1	40.59	24.76	62	616	0	976.86	10.75	52	35	male	LJU	S
13	16	16	24	26.6	99	0	0	0	0	2340	21.1	40.46	27.26	55	617	0	976.87	10.77	52.4	35	male	LJU	S
14	17	18	24	26.6	98	0	0	0	0	2400	21.1	40.36	37.63	48	585	0	976.87	10.77	52.5	35	male	LJU	S
14	18	20	23.9	26.6	99	0	0	0	0	2460	21.1	40.45	55.98	43	555	0	976.82	10.94	55	35	male	LJU	S
14	18	19	23.9	26.6	99	0	0	0	0	2520	21	40.46	19.78	39	502	0	976.9	10.91	54.5	35	male	LJU	S
13	14	16	23.9	26.6	99	0	0	0	0	2580	21	40.56	16.24	36	512	0	976.87	10.84	53.4	35	male	LJU	S
13	19	19	23.9	26.6	97	0	0	0	0	2640	21	40.59	16.66	32	555	0	976.91	10.87	53.9	35	male	LJU	S
15	19	20	23.8	26.6	98	0	0	0	0	2700	21	40.57	30.64	29	525	0	976.86	10.89	54.2	35	male	LJU	S
NA	NA	NA	NA	NA	NA	NA	NA	NA	NA	NA	21	40.61	47.72	27	504	0	976.87	10.21	43.6	35	male	LJU	S
15	20	20	23.8	26.6	99	0	0	0	0	2760	21	40.71	15.63	25	546	0	976.82	10.83	53	35	male	LJU	S
