# Supplementary material for: Harmonization and Visualization of Data from a Transnational Multi-Sensor Personal Exposure Campaign
Source: Int J Environ Res Public Health. 2021 Nov 4;18(21):11614. doi: 10.3390/ijerph182111614 (PMC8583633; doi:10.3390/ijerph182111614)
Supplement: Supplementary file 1 [file ijerph-18-11614-s001.zip › SD-C.pdf]

Personal exposure to air pollutants - ICARUS project report to participant

Dear “XY”,

Thank you for your participation in the ICARUS sampling campaign (https://icarus2020.eu) aiming to characterize your personal exposure to air pollutants by using several personal sensors (activity tracker, portable particulate matter sensor device, silicon wristband) and a static sensor (uHoo) placed in your household. As per our liability given by GDPR, we are providing you ‘your’ personal data, analysed in the form of report below. Even though it is difficult to explain complex scientific terms in simple language, we have used simple and easy language and graphics to convey information. Enclosed you will find your personal data in the form of graphical representations of results gathered by personal and static sensors.

This report is comprised of three parts: Part A with an overview of general objectives of the campaign and parameters measured, Part B with results from your household, and Part C with your personal exposure data.

When examining your report, please pay attention to the trends and values which fluctuate during the day and between the seasons. Due to individual daily routines, characteristic for each participant, you will most likely notice patterns in your daily tasks and more easily follow the recommended actions to reduce your exposure, which can be found at the end of this report. We sincerely thank you for your participation and hope to see you take part in similar campaigns in the future.

With warm regards,

On behalf of the ICARUS Ljubljana team,

Dr. XY

Part A – Introduction of the campaign

ICARUS sampling campaign was conducted in the following European cities: Athens, Basel, Brno, Madrid, Milan, Ljubljana and Thessaloniki. The aim was to recruit participants from all ages and sociodemographic backgrounds to gather information about their exposure to air pollutants. The process included both at home and personal monitoring for seven days, in both summer and winter periods. The main objectives of the campaign were: -Collect data on external environmental exposure and exposure determinants by combining location, activity and air pollution data in different microenvironments, - Demonstrate feasibility of using new sensor and mobile technologies in collecting exposure data, - Analyse and compare exposure data in several different European cities. The types of information were collected using following devices/means: - Exposure monitoring devices: (i) static "uHoo" sensor measuring temperature (T), relative humidity (RH), Particulate matter (PM), Volatile Organic Compounds (VOC), air pressure (AP), carbon dioxide (CO_2), carbon monoxide (CO) and nitrogen dioxide (NO_2) in indoor environments; (ii) portable particulate matter (PPM) sensor measuring three PM sizes (<1, <2.5 and <10 um), temperature, humidity and GPS location; - Physical activity tracker: Garmin Vivosmart 3 Activity Meter to monitor activity and fitness level. The device monitors heart rate, steps, calories burned, sleep and other activities. - Questionnaires to collect additional information on living environments, habits, socio-economical information of individuals, such as: age, household type, individual and family income, underlying health conditions, etc. - Time activity diaries where users recorded their daily activities in one-hour resolution.

When reading and interpreting the results provided in the next sections, the following should be kept in mind: - Use of new sensing technologies in an extent as it was done within the ICARUS project, resulted in a number of technical issues with some devices during the campaign, resulting in data gaps for some participants. - While static devices are able to capture changes and trends in distribution of air quality (AQ) parameters measured, they suffer from too high uncertainty for absolute values to be used. To this end, absolute values are given for T and RH only, and differences in AQ parameter over time are indicated in a form of heat-maps showing a colour scale corresponding to the lowest and highest values of that specific AQ parameter. - In some cases, time activity diaries (TAD) do not provide information with temporal resolution detailed enough and consequently measured AQ parameters do not necessarily fully correspond to activity indicated in the TAD.

Moreover, to help with the interpretation, in Appendix A, on page 10, you can find basic information on measured and reported parameters: their general characteristics, main sources, health effects, and options for reducing exposure to selected air pollutants.

Part B – Situation in your household

Important note: In case you will not find charts and tables populated for one of your seasons, the reason might be technical problems encountered or the fact that you were participating during one season only. ### Meteorological parameters In Figure 1 meteorological conditions (temperature, relative humidity and air pressure) in your household for the whole sampling period and for both seasons are shown.

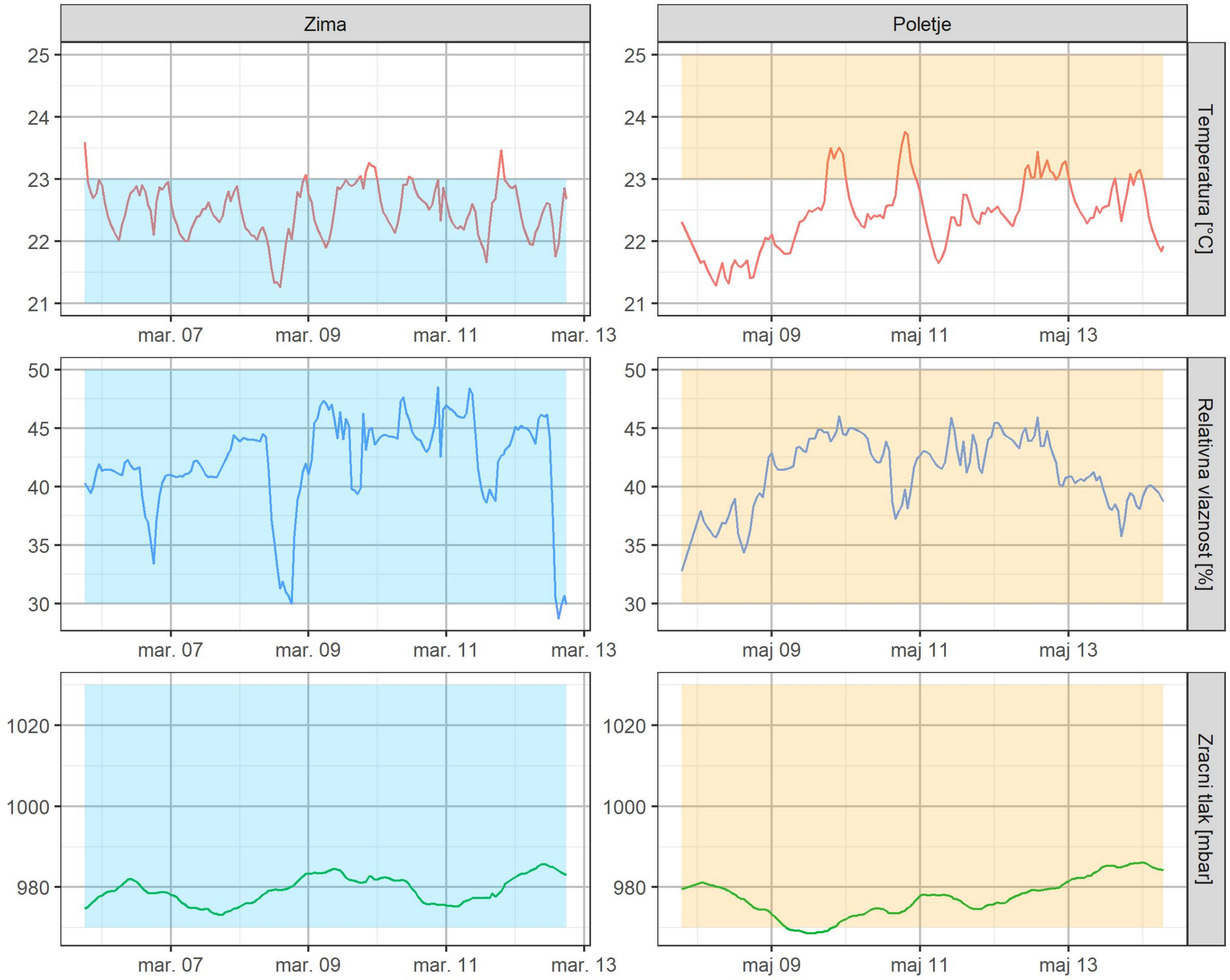

Figure 1: Meteorological conditions in your household during the winter (left) and summer (right) campaigns. The top plot displays temperature, followed by relative humidity and air pressure. Optimal ranges for all three parameters are also displayed and coloured in yellow (summer) and blue (winter).

Air quality parameters

In Figure 2 air quality parameters (CO_2, NO_2 in TVOC) in your household for individual days in both seasons are shown. During the campaign, CO was not detected, and was left out of this report. To interpret the observed distribution and changes of individual parameters over time comparison with activity reported in the time activity diary for each individual day is needed.

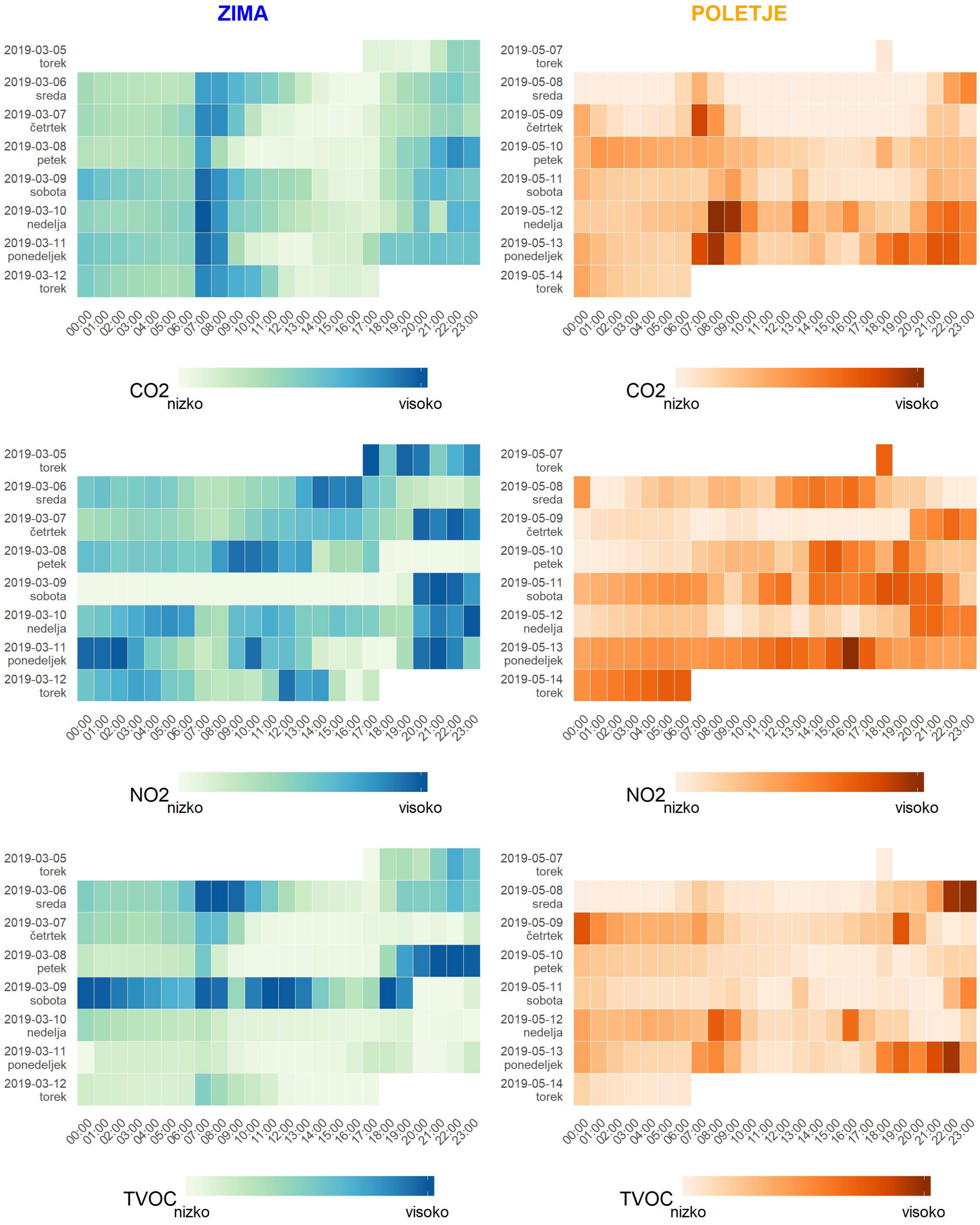

Figure 2: Air quality parameters (CO_2, NO_2 in TVOC) in your household for individual days in both seasons (winter and summer) are shown in a form of heat-maps displaying relative deviation of each parameter in comparison to highest and lowest measured values in winter (left) and summer (right) measuring campaigns. The label "low" represents the lowest measured value, while the label "high" the highest measured value. It is not possible to directly compare the winter and summer charts, as the displayed values are relative.

Part C – Personal exposure to air pollutants

In this section results on personal exposure obtained by personal particulate matter (PPM) sensor and Garmin Physical Activity (PA) tracker are given.

Exposure to particulate matter

In Figure 3, concentrations of PM_1, $PM_{2.5}$ and PM_{10} and heart rate, obtained by portable sensor devices for the whole sampling period and both seasons, are shown as the original minute resolution data in order to emphasise variations in concentrations over time. Line breaks in the charts indicate data gaps mentioned above.

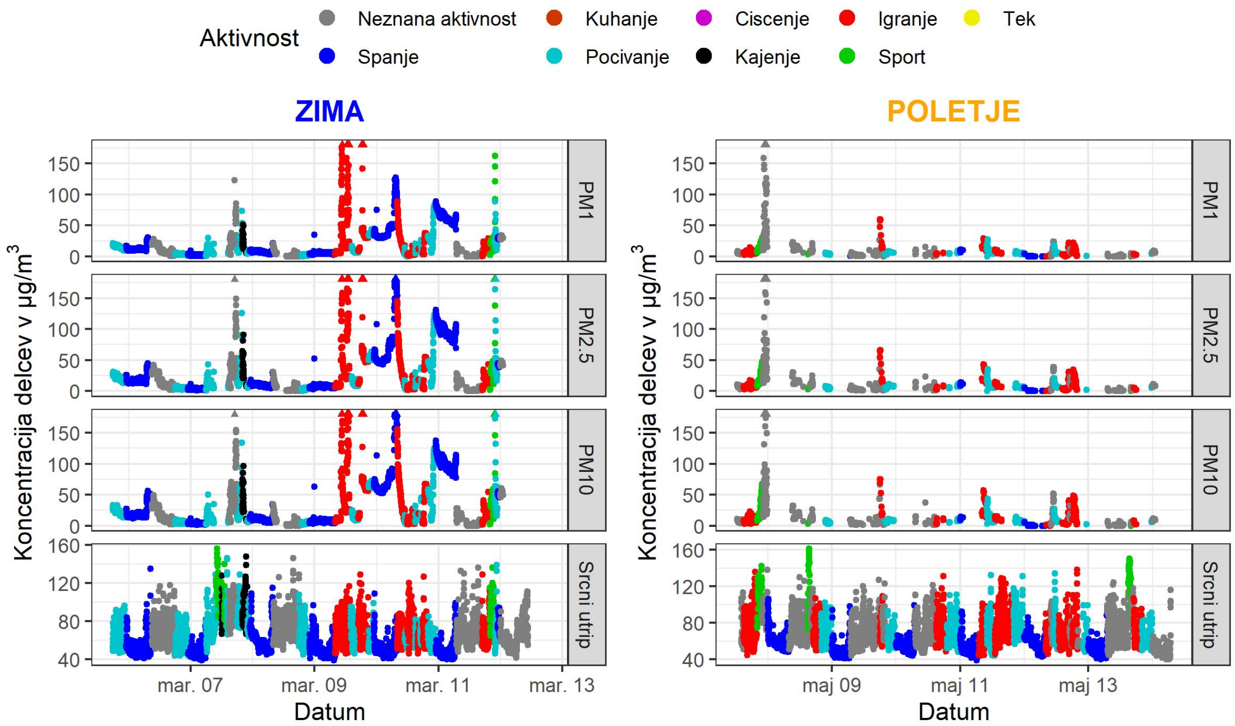

Figure 3: Measured concentrations of particulate matter (PM) in different sizes (<1, <2.5 and <10 um displayed as PM 1 , PM 2.5 and PM 10) as well as heartbeat in winter (left) and summer (right) together with activity data. Grey dots indicate a period with unknown activity, during which the PM concentrations and/or heart beat might still be known.

In Figure 4, daily averages of PM data collected from the PPM device are compared with World Health Organization (WHO) Air Quality Guideline values which set 24-hour means for $PM_{2.5}$ at 25 ug/m^3 and for PM_{10} at 50 ug/m^3.

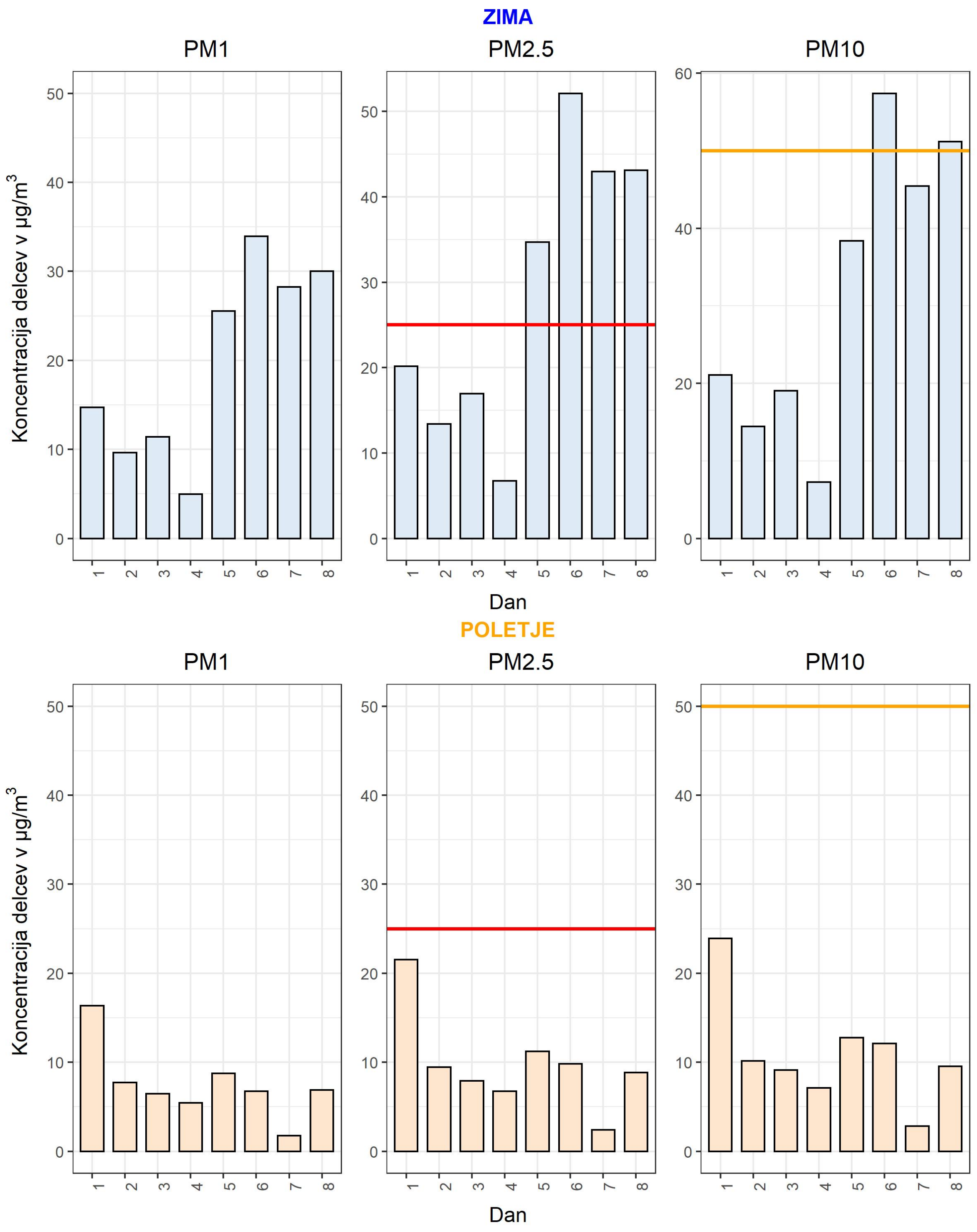

Figure 4: Comparison of average daily PM values with WHO Air Quality Guideline values for winter (upper chart) and summer campaigns (lower chart). The red line represents the WHO guideline for 24-hour average value for PM 2.5 and the orange line represents the WHO guideline for 24-hour average concentration of PM 10 . No guideline values exist for PM 1 . Days on the x axis (horizontal axis) represent the number of days the person participated in each season. A day without a column means the person participated that day, but no PM data was recorded. The displayed averages are only indicative as they depend on the availability of the data (Figure 3).

Physical activity

In Table 2 a summary of physical activity data during both seasons is given. Values correspond to daily values (00:00-23:59), while PA parameters are defined as follows (source: Garmin Vivosmart 3 user manual): - Steps: The total number of steps per day. - Stress level: Calculated based on heart rate variability, the stress level range is from 0 to 100, where 0 to 25 is a resting state, 26 to 50 is low stress, 51 to 75 is medium stress, and 76 to 100 is a high stress state. - Average heart rate (HR): Average number of heart beats per minute. - Maximum HR: Maximum daily hearth rate. - Sleep time: Total hours of sleep. - Calories: The amount of total calories burned during the day, including both active and resting calories.

Table 1: Summary of physical activity data during both seasons (upper table – winter, bottom table - summer). The value "0", NaN" and "-Inf" means a specific data is not available.

Dan	Koraki	Stopnja stresa	Povprecen srcni utrip	Najvisji srcni utrip	Ure spanja	Kalorije
1	1327	NaN	61	96	1	35
2	8250	12	58	135	8	255
3	11396	50	77	156	6	845
4	9158	24	66	146	7	349
5	7547	19	60	129	9	242
6	8587	14	59	127	9	184
7	11663	28	64	139	7	463
8	1594	20	61	111	6	80

Dan	Koraki	Stopnja stresa	Povprecen srcni utrip	Najvisji srcni utrip	Ure spanja	Kalorije
1	6468	20	84	142	0	644
2	10342	20	67	161	8	713
3	8724	14	62	127	7	296
4	9330	14	65	131	7	396
5	13554	16	67	132	7	590
6	6236	15	62	138	8	245
7	15673	15	64	150	7	840
8	112	6	48	116	7	24

Average PM values per activity

Average PM values per activity are displayed on the plot and enable a direct comparisons between activities and seasons. However, pay close attention to the y-axis (vertical), as the values are displayed in different scales.

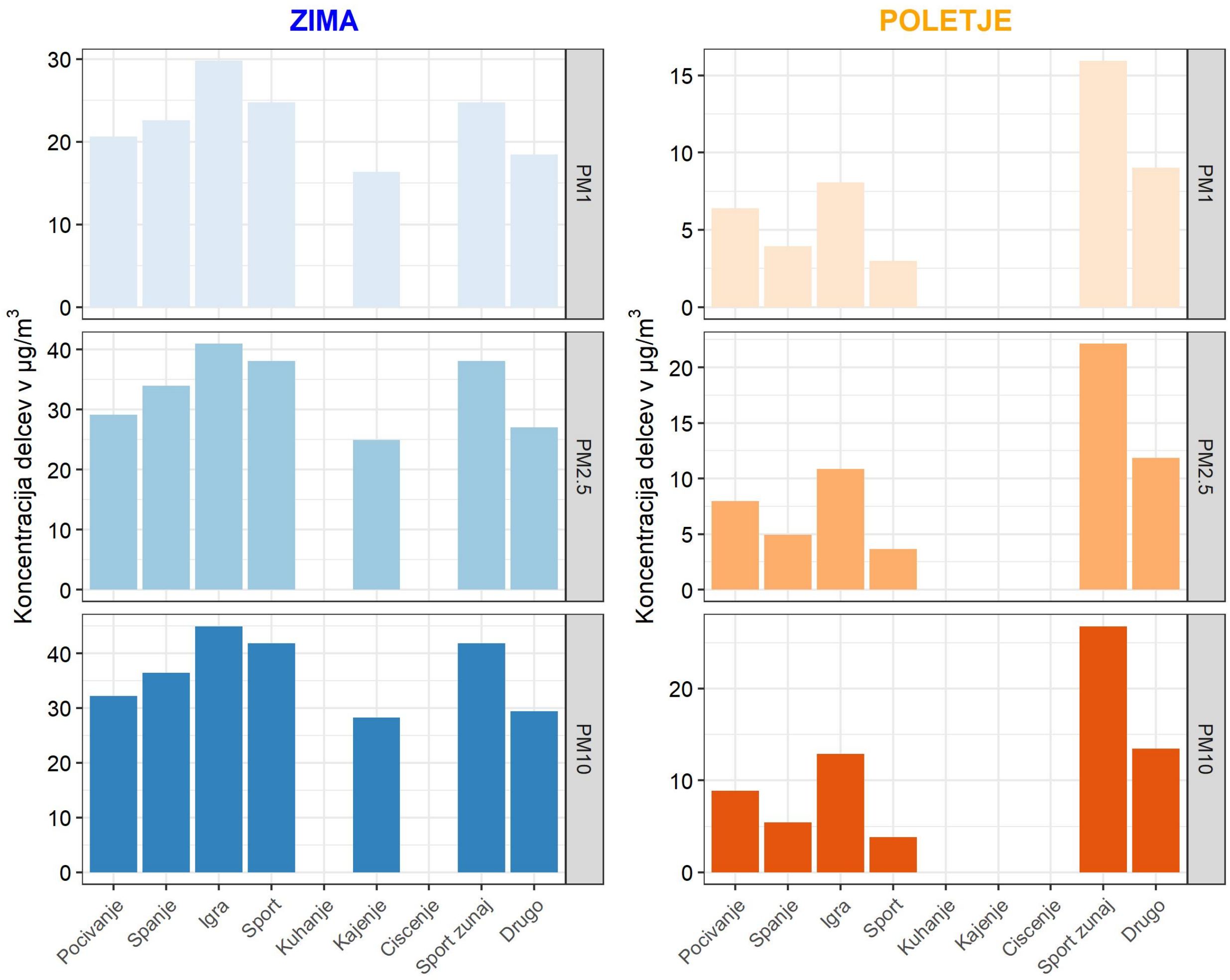

Figure 5. Average PM values per activity of the person during winter (left) and summer (right). Activity without a histogram pillar means that you did not undertake those activities during your participation. The displayed averages are only indicative as they depend on the availability of the data (Figure 3).

Simple suggestions to reduce harmful effects of air pollution

How to improve indoor air quality?

- Ventilate often and properly,
- Ventilate bathroom after taking a shower,
- Ventilate during and after cooking,
- Ventilate after using the fire place,
- If you live next to a highly trafficked road, ventilate fast and avoid ventilating during rush hours,
- Do not smoke indoors,
- Do not use candles, incense sticks etc.,
- Do not overuse detergents and disinfectants,
- Choose cleaning products and paints which do not contain VOCs,
- Do not save opened paints etc. around,
- Choose natural materials and avoid chipboard,
- Air out VOCs from your new furniture outdoors or in a well-ventilated room,
- Get rid of unnecessary upholstery and carpets,
- Change and clean the filters in your air conditioner regularly,
- Dust 1-2 times a week,
- Clean the fire place after the heating season,
- Craft materials (e.g. glue) do not belong in the living room,
- Keep the room temperature and relative humidity within the optimal ranges

Appendix A: General information on parameters reported (source: uHoo)

Param.	General info/sources	Potential negative effects	What to do?
**T**	Temperature affects the body and there is an optimal range for people to feel comfortable and productive. 21 °C - 23 °C is the optimal range for human occupancy during winter, and 23 °C -25°C during– summer.	Going outside of the optimal range can make a person uncomfortable, and mental and physical abilities may be impaired. In addition, setting the room temperature too low can increase the risk of mold due to the cold air transporting water vapor around, increasing the amount of condensation on furniture, walls and other surfaces.	Adjusting the air vents and controlling the HVAC system are still the best ways to control temperature in the home. Getting a smart thermostat and/ or air sensor system to control the temperature also makes It easier and ensures the temperature is always optimal.
**RH**	Relative humidity (RH) refers to the amount of water vapor present in the air. The humidity rating is given as a percentage. The ideal range for humidity indoors is between 30 % to 50 %.	Humidity is important as humans depend on perspiration to regulate their body temperatures, and high humidity affects this process reducing the rate of evaporation of moisture from the skin.	If your home suffers from high humidity, avoid drying laundry indoors. Using air conditioning dries out the air and removes moisture. When cooking and bathing, ensure that exhaust fans are on or a window is open to bring fresh, dry air inside. If the air is too dry you can hang wet towels over a radiator.
**AP**	Air pressure, also known as barometric pressure, refers to the pressure air exerts on everything in a room. High air pressure means the air is usually still and can allow the buildup of pollutants in the room. 970-1030 mbar is considered normal and positively influences air flow and human well-being.	Excessive air pressure can negatively influence people and the amount of air that flows in and out of the room. It's best to match the air pressure of the ambient air outside your home.<br><br>Increased air pressure can cause headaches and migraines, and even affect blood pressure. Those with joint paint or issues with balance know that high AP can make it worse.	Make sure that exhaust fans are properly balanced. Too many exhaust fans can remove too much air in the house and create negative air pressure. Ducts and ventilation should be properly designed to avoid compressing the air, leading to positive air pressure.
**CO_2**	Carbon Dioxide (CO_2) is one of the many common molecules found in the air. It is usually emitted by humans through the breathing process, but can also be created through various forms of combustion such as cooking and the use of automobiles.	It is harmless in small quantities but can have increasing health effects as concentration levels rise. Too much CO_2 in the indoor air can cause headaches and other problems such as drowsiness or restlessness and has been associated with lowered productivity and illness.	Proper ventilation is the best way to control CO_2 levels, so open the windows regularly to allow fresh air inside.

**VOC**	Volatile Organic Compounds (VOCs) refer to organic compounds which easily become vapor or gas emitted from a wide variety of everyday products. These include solvent based paints, air sprays, scent products, adhesives, and more. They are commonly introduced into the home through newly-bought furniture and linens, office equipment along with aerosol sprays, chemical cleaners and disinfectants etc.	VOC concentration tends to be ten times higher indoors than outdoors and can present health issues.<br><br>Short term exposure can result in headaches, nausea, dizziness, and irritation of the respiratory tract and the eyes. Long-term exposure (months to years) to high levels of VOC can cause liver damage, kidney damage, and cancer.	Recognizing the sources of pollution in your home is the first step. Most VOCs come from sources that can be avoided or substituted with less polluting ones. Secondhand furniture for example emits much less VOC than new one.<br><br>Use natural cleaners like vinegar, lemon juice or baking soda instead of chemical sprays. If you have any new furniture or linens, air them out outdoors before using them.
**NO_2**	Nitrogen Dioxide (NO_2) is a red-brown gas that is released when fuel burns. It is most often found in automobile exhausts, and the fumes from burning fossil fuels such as propane, kerosene, natural gas and wood.	In high concentrations, it can irritate airways while long-term exposure can result in chronic illness and respiratory infections.	The main sources are unvented stoves and appliances, so ensure that these are property sealed or vented to avoid leaking into the home. Tobacco smoking is also a major source of NO_2, along with kerosene lamps and heaters.
**CO**	Carbon Monoxide (CO) is a byproduct of the combustion of fossil fuels, and common sources include tobacco smoke, heaters that use fossil fuels, central heating furnaces and, automobiles.	CO is a highly toxic indoor air contaminant that can be very dangerous to human health. It is a colorless, odorless gas that can cause health damage in very short spans of time.	Ensure that gas appliances are properly sealed and gas boiler rooms well ventilated. Open the flue over a fireplace when in use. Do not leave a car idling in the garage. Any tools or equipment that use gas should be operated outdoors. Install CO sensor for your safety.
**$PM_{2.5}$**	$PM_{2.5}$ refers to particulates that have a diameter of less than 2.5 micrometers, and are known as fine particles. The main sources indoors are pet dander, molds, fossil fuel burners (like the stove), and chemicals from cleaning supplies.	These particles are especially dangerous due to their small size, which means they stay aloft in the air longer, increasing the chance for contact with them. In addition, their smaller size makes them easily inhalable, and they can get into the lungs and even the bloodstream.	$PM_{2.5}$ can be reduced in various ways: When cooking, make sure that the room is well-ventilated, don't let your pets in the bedroom, wipe the dust regularly and avoid excess items that can collect dust, When cleaning use natural solutions like a mixture of vinegar or lemon juice.
